# Neurobiological alteration in agitation in Alzheimer’s disease and possible interventions

**DOI:** 10.3389/fpsyt.2024.1412901

**Published:** 2024-07-09

**Authors:** Jagadeesh S. Rao, María Alejandra Tangarife, Ram Mukunda

**Affiliations:** Science Department, India Globalization Capital (IGC) Pharma, Potomac, MD, United States

**Keywords:** agitation, Alzheimer’s disease, neuroinflammation, GABA, CBR-1, THC, melatonin

## Introduction

The presence of agitation in dementia patients with Alzheimer’s Disease (AD) is a complex behavioral phenomenon that arises as a result of the progression of the disease. According to reports, the prevalence of agitation in mild cognitive impairment and AD is 60% and 76%, respectively (1). The exact cause of agitation in AD remains unclear; however, various pathological cellular events have been linked to it. These include the upregulation of neuroinflammation in specific regions of the brain (2), the involvement of inflammasomes -3 (3), the reduction of CB1 receptor (CB1r) functionality, and imbalances in brain neurotransmitters (4). The development of therapeutic approaches that target these hallmarks suggests the potential effect of reducing agitated behavior in clinical settings. This opinion will explore agitation in AD by describing the molecular basis of agitation and how therapeutic agents THC and melatonin reduce agitation by targeting neuroinflammation, CB1r imbalance, and neurotransmitter imbalances.

## Agitation arising from specific brain regions

Recent positron emission tomography (PET) studies in AD patients have demonstrated that the upregulation of inflammation in the brain’s frontal and medial temporal regions is associated with agitation behavior in AD patients (2). Research has explored the mechanism underlying the onset of agitation, revealing associations with structural and functional deficits in key brain regions, including the frontal cortex (FC), the anterior cingulate cortex (ACC), the posterior cingulate cortex (PCC), the insula, and the hippocampus (5). These structures are associated with cognitive processes, decision-making, motivation, emotion processing, memory, and spatial navigation (6). CB1r is essential in regulating emotional behaviors, such as anxiety and fear, and cognitive functions, such as memory (7). The brain regions associated with agitation and other behaviors within the spectrum of hyperactivity syndrome (8) are rich in the CB1 receptors gene and protein expression (9), suggesting the role of endocannabinoids in regulating these behaviors. Equally important, the damage in these same regions is associated with hyperarousal in insomnia patients (10). In AD, aggressive behaviors have been also linked to circadian disturbance and the melatonin system (11).

## Neuroinflammation regulation by cannabinoids

The endocannabinoid system is a crucial player in neuroinflammation regulation. This system is composed of two G-proteins coupled cannabinoid receptors, CB1r and CB2r. Endogenous cannabinoids, such as anandamide (AEA) and 2-arachidonoylglycerol (2-AG), are produced from lipid precursors on demand and act as retrograde transmitters in neuron-to-neuron communication as well as mediate neuron-glia interaction. They are also involved in intracellular signaling that regulates mitochondrial activity (12). CB1r, the most prevalent receptor in the brain, and is primarily found in neurons. In contrast, CB2r expression is minimal, and it is primarily found in glial cells. The local CB1r activation in the hippocampus is crucial for sustaining neurogenesis, mitigating neuroinflammation, and averting cognitive impairment (12). The loss of CB1r in the brain may lead to elevated neuroinflammation due to the disruption of CB1r’s communication with CB2r (12).

## Neurotransmitter imbalance stability by cannabinoids

The endocannabinoid system plays a significant role in regulating neurotransmitter release and their receptor function. Studies have revealed that deficits in the GABAergic and serotonergic functions are associated with agitation in AD (4). Specifically, reduced GABA levels/transmission and altered serotonergic system functions, which are linked to the loss of serotonergic neurons in the raphe nuclei and their projections to the cortex (mainly PFC) and the amygdala may be implicated in associated agitation behavior in AD (4, 13, 14). In normal conditions, the GABA/Glutamate ratios are inversely correlated in the salience network (SN) and the default mode network (DMN) of the brain, suggesting that the levels of GABA in the SN play a role in the resting state functional connectivity of the SN and its interactions with the DMN (15). The SN contributes to various brain functions such as communication, social behavior, and self-awareness, which is possible through the integration of sensory, emotional, and cognitive information, and the DMN refers to areas in the brain activated when people’s mind wander at rest (16). Research suggests that in pathological conditions, the SN excitatory/inhibitory interactions get dysregulated, as reflected by the reduction of the GABAergic inhibitory signaling (17) and the dysregulation of serotonin function (18). Studies have shown that cannabinoid agonists stimulate [3H]GABA release by activating the CB1r in rats (19). Additionally, they have also been shown to impair the functionality of 5-HT1A and 5-HT2A/C receptors CB1r knockout mice (18). These findings suggest that the activation of CB1r using CB1r agonist will regulate GABA release and stabilize the serotonergic receptor functionality without affecting the 5-HT levels. CB1r partial agonist, THC, may provide a therapeutic effect against agitation.

## Melatonin role in aggressive behavior

Melatonin is a neurohormone synthesized in the pineal gland from its precursor serotonin. Several animal studies have supported the idea that melatonin or melatonin receptor agonists reduce aggressive behaviors in various animal paradigms (11). Elderly persons have decreased levels of melatonin in CSF, blood, saliva, and urine, and patients with AD may even have more declined levels of melatonin than control subjects (20). This may be due to the reduction in functional pineal gland volume and suprachiasmatic nucleus cells (20). The clinical studies have shown mixed results on the efficacy of melatonin on agitation due to variability in dosing (11).

## Role of inflammasomes-3 in aggressive behavior and inhibition by THC and melatonin

Chronic neuroinflammation has been shown to be a major factor in the pathophysiology of AD by several studies (21). One of the most important molecular connections in the AD neuroinflammatory pathway is the nucleotide-binding oligomerization domain-like receptor pyrin domain-containing 3 (NLRP3) inflammasome (21). The NLRP3 inflammasome-driven inflammatory response has been implicated in aggressive behavior in animals (3). An animal study demonstrated that the NLRP3 inflammasome-driven inflammatory response contributed to resident intruder paradigm-induced aggressive behavior in mice (3). Both CB1r partial agonist THC (22) and melatonin (23) inhibit inflammasome -3 *in-vitro* models. These studies imply that both THC and melatonin attenuate inflammasome activity and bring beneficial effects on aggressive behavior, suggesting a potential path for therapeutic intervention.

## Protection against aggressive behavior by CB1r agonist

CB1r knockout mice display aggressive behaviors, but the administration of the CB1r agonist Arachidonyl-2’-chloroethylamide (ACEA; 2 mg/kg) to these mice has been shown to decrease their aggression behavior significantly (24). These results suggest the involvement of CB1r in aggressive behavior and social interaction (24). The loss of CB1r has been linked to aggressive behavior in CB1r knockout mice, but research suggests that this behavior was corrected with acute administration of CB1r agonists (24). It is also worth noting that reduced hippocampal CB1r activity was reported in AD patients (25).

## Decrease in agitation behavior in AD by THC and melatonin

Several off-label studies on synthetic THC and medicinal cannabis oil containing THC (26–28) have demonstrated a significant reduction in agitation behavior in AD. A study comprising 17 melatonin trials that ranged in dosage from 3 to 10 mg over 10 days to 35 months showed improvement in agitation behaviors in AD (29). It concluded that longer trials on melatonin duration had the most improvement in agitation behaviors, further reinforcing confidence in the potential of these therapeutic agents. Several off-label antipsychotic drugs and the recent FDA-approved brexiprazole drug have been used to treat agitation in AD, however, the complete mode of action is not clear (30). The effects of brexpiprazole on serotonin and dopamine receptors are considered to be the mechanism of action (31). Brexpiprazole exhibits partial agonism on some dopamine and serotonin receptors while antagonistically acting on other serotonin receptors, suggesting that its receptor selectivity is advantageous (31). Combinational therapy of THC and melatonin for agitation behavior provides a synergetic effect on neurobiological pathways and may bring out early therapeutic effects.

## Discussion

**Figure 1:**
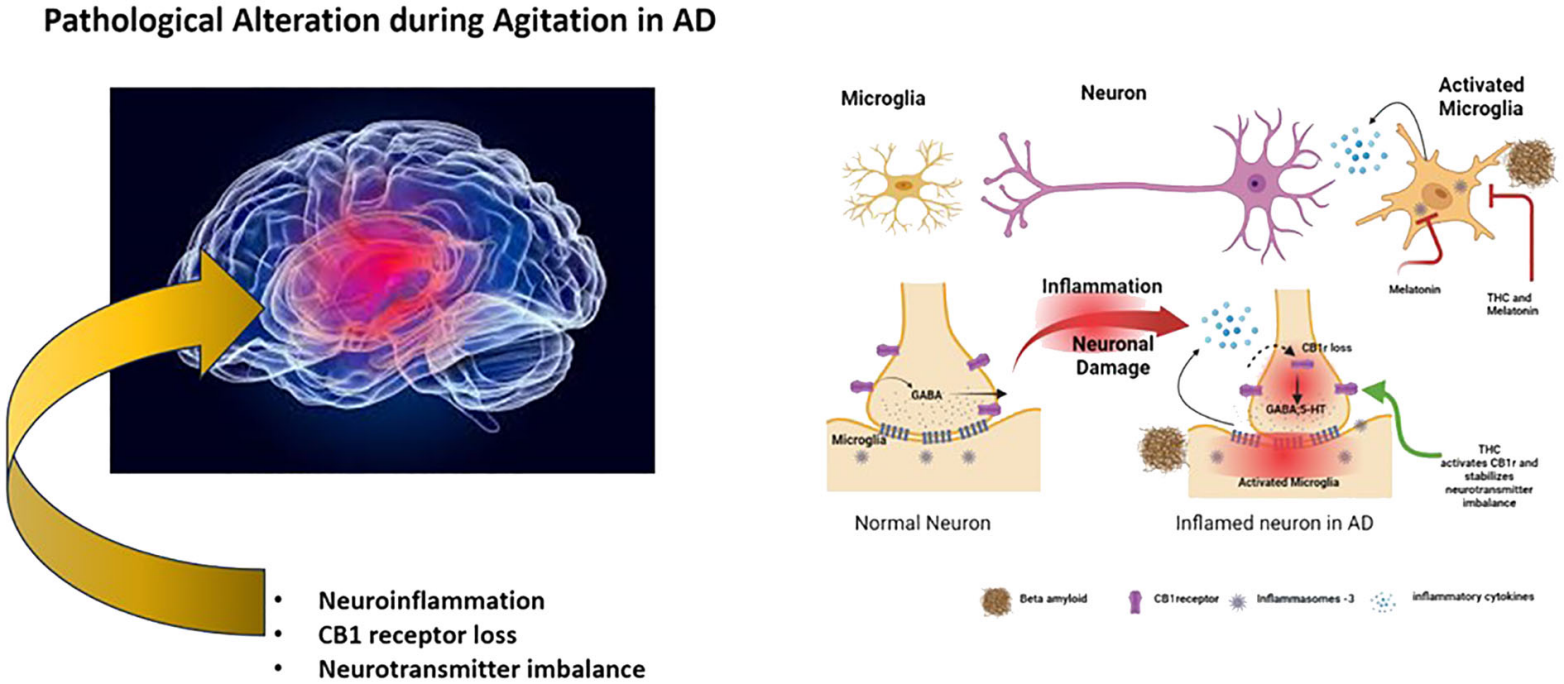
Schematic representation of pathological alterations in agitation of AD and its intervention by THC and melatonin.

The proposed action of THC and melatonin displays a synergistic effect on mitigating various neuroinflammatory pathways, neurotransmitter imbalances, and CB1r imbalances, all hallmarks implicated in the development of agitated behavior in the Alzheimer’s brain (**Figure 1**). The potential of this combination is expected to stabilize mood, as seen in its effect on GABA levels by both THC and melatonin (19, 32). In support of this notion, a case study with Gabapentin showed positive results that suggested the reduction of agitation behavior in AD (33). However, these assertions are constrained by certain limitations, such as a non-randomized trial design, a limited sample size, and the lack of placebo-controlled studies. Accumulating evidence supports the role of GABA as an anti-inflammatory agent (34), further indicating a more indirect effect on the reduction of agitation as promoted by the combination of THC and melatonin. As reported in previous experiments with animal models, the administration of CB1r agonists has also reported decreased levels of aggression (24), suggesting a positive effect for treating agitation in AD. The combination with melatonin will likely display longer-term reductions of agitation, opening new possibilities for therapeutic interventions. In line with this notion, there are seven cannabis-based trials related to dementia that are currently listed on clinicaltrials.gov. One is on general neuropsychiatric symptoms, while the other six are on agitation. The compounds used in the experiments include synthetic THC, CBD, THC-CBD, and a THC+CBD+CBC combination (35). As research in this field continues to evolve, the synergistic effects observed between THC and melatonin may present them as potential candidates for developing treatments that could significantly contribute to the management of mood-related disorders. Different pathways may be involved in bringing out the beneficial effects that THC and melatonin target, and more extensive placebo-controlled studies are warranted to determine the efficacy of combinational therapy of THC and melatonin, which will be critical when including the effect of metabolism. The cytochrome 2C9 enzyme is polymorphic and is responsible for the most significant metabolism of THC (36). Since cytochrome 2C9 is polymorphic, it impacts the effectiveness of THC and should be carefully titrated when administering THC dosages. In a recent pilot study on the safety and tolerability of multiple ascending doses of THC and melatonin in patients with AD, no serious adverse events or deaths were reported. However, a larger sample study is needed to fully understand the long-term effects of THC and melatonin on potential adverse events on these groups (37). The development of therapeutic agents containing CB1r agonist and melatonin may provide an efficient therapeutic effect in treating agitation in AD patients. Further trials are warranted to examine their effect on these unmet AD behavioral conditions.

## Author contributions

JR: Conceptualization, Data curation, Formal analysis, Investigation, Resources, Writing – original draft, Writing – review & editing. MT: Writing – review & editing. RM: Conceptualization, Writing – review & editing.
